# A Peptide Mimetic of 5-Acetylneuraminic Acid-Galactose Binds with High Avidity to Siglecs and NKG2D

**DOI:** 10.1371/journal.pone.0130532

**Published:** 2015-06-25

**Authors:** Laura L. Eggink, Georgios A. Spyroulias, Norman G. Jones, Carl V. Hanson, J. Kenneth Hoober

**Affiliations:** 1 Susavion Biosciences, Inc., Tempe, AZ, 85281–3257, United States of America; 2 Department of Pharmacy, University of Patras, GR-26504, Patras, Greece; 3 Viral and Rickettsial Disease Laboratory, California Department of Public Health, Richmond, CA, 94804, United States of America; Universidade de São Paulo, BRAZIL

## Abstract

We previously identified several peptide sequences that mimicked the terminal sugars of complex glycans. Using plant lectins as analogs of lectin-type cell-surface receptors, a tetravalent form of a peptide with the sequence NPSHPLSG, designated svH1C, bound with high avidity to lectins specific for glycans with terminal 5-acetylneuraminic acid (Neu5Ac)-galactose (Gal)/N-acetylgalactosamine (GalNAc) sequences. In this report, we show by circular dichroism and NMR spectra that svH1C lacks an ordered structure and thus interacts with binding sites from a flexible conformation. The peptide binds with high avidity to several recombinant human siglec receptors that bind preferentially to Neu5Ac(α2,3)Gal, Neu5Ac(α2,6)GalNAc or Neu5Ac(α2,8)Neu5Ac ligands. In addition, the peptide bound the receptor NKG2D, which contains a lectin-like domain that binds Neu5Ac(α2,3)Gal. The peptide bound to these receptors with a K_D_ in the range of 0.6 to 1 μM. Binding to these receptors was inhibited by the glycoprotein fetuin, which contains multiple glycans that terminate in Neu5Ac(α2,3)Gal or Neu5Ac(α2,6)Gal, and by sialyllactose. Binding of svH1C was not detected with CLEC9a, CLEC10a or DC-SIGN, which are lectin-type receptors specific for other sugars. Incubation of neuraminidase-treated human peripheral blood mononuclear cells with svH1C resulted in binding of the peptide to a subset of the CD14^+^ monocyte population. Tyrosine phosphorylation of siglecs decreased dramatically when peripheral blood mononuclear cells were treated with 100 nM svH1C. Subcutaneous, alternate-day injections of svH1C into mice induced several-fold increases in populations of several types of immune cells in the peritoneal cavity. These results support the conclusion that svH1C mimics Neu5Ac-containing sequences and interacts with cell-surface receptors with avidities sufficient to induce biological responses at low concentrations. The attenuation of inhibitory receptors suggests that svH1C has characteristics of a checkpoint inhibitor.

## Introduction

An extensive number of lectin-type cell-surface receptors regulate activity of immune cells [1]. Some are C-type lectins, which bind sugars in a calcium-dependent manner [2,3]. A C-type galactose (Gal)/N-acetylgalactosamine (GalNAc)-binding receptor, MGL/CD301/CLEC10a, is expressed on the surface of immature dendritic cells and macrophages and is involved in endocytosis [3–5]. Other examples of C-type lectins that undergo endocytosis include DC-SIGN/CD209, a mannose (Man)-binding receptor on dendritic cells and macrophages; MRC1/CD206, a Man receptor on macrophages; Langerin/CD207, a high Man and galactose-6-sulfated oligosaccharide receptor on Langerhans cells [6,7]; and Dectin-1/CLEC7a, a β-glucan receptor on macrophages [1].

Another large family of glycan-specific receptors includes I-type lectins that belong to the immunoglobulin superfamily. The best characterized members of I-type lectins are siglecs (sialic acid-binding immunoglobulin-like lectins), which bind sialic acid (5-acetylneuraminic acid, Neu5Ac)-containing glycans and modulate cellular signaling events and maturation of immune cells [8–12]. The siglec family in humans comprises 14 different proteins expressed on various cells of the immune system [11,12]. The cell surface is abundantly decorated with sialylated glycans and thus these receptors can bind glycan ligands on the same cell (*cis*) or on neighboring cells (*trans*) [13]. Upon binding of ligands, siglecs are phosphorylated and undergo endocytosis [11–15]. Most siglecs contain one or more inhibitory immunoreceptor tyrosine-based inhibitory motifs (ITIMs) in their cytoplasmic domain [9–12]. The negative regulation of T cells by Siglec-7 and Siglec-9 requires ligand binding for optimal activity [16]. In contrast, Siglec-14, -15 and -16 lack the inhibitory domain and function in conjunction with an activating adaptor protein, DAP12, which contains an immunoreceptor tyrosine-based activation motif (ITAM) [11,12,17,18].

To study ligand interactions with these receptors, a series of Neu5Ac-containing glycans were synthesized by chemical and enzymatic reactions [19] that bound to siglecs with affinities in the millimolar range [20,21]. Multivalent structures have increased avidity that provides binding constants in the micromolar range [21–23]. As an example, Siglec-5 binds α(2,3)- or α(2,6)-linked sialosides with K_D_ values of 8.7 and 8.0 mM, respectively, with a K_D_ for α(2,8)-linked disialic acid of 25 mM [23]. However, multivalent, polyacrylamide-conjugated sialosides with α(2,3)- or α(2,6)-linkages were bound with a K_D_ of 2 to 4 μM, whereas the K_D_ for α(2,8)-linked multivalent disialic acid structures was 0.4 μM [23].

Some receptors may have non-glycan ligands *in vivo* yet contain an extracellular lectin-like domain. The receptor NKG2D on natural killer (NK) cells, γδ T cells and CD8^+^ cytotoxic T cells is regulated by endogenous polypeptide ligands such as MICA/B, ULBP, Rae-1 or H60 [24–26], but NKG2D also contains a lectin domain adjacent to the polypeptide binding site that binds Neu5Ac(α2,3)Gal- sequences [27].

Because siglecs are important regulators of the immune system, ligands with high affinity should provide valuable tools to address therapeutic opportunities [11,12,28]. A question of interest is how to design ligands that bind to these regulatory receptors with sufficient avidity and specificity to achieve manipulation of the immune system. To explore this possibility, we asked whether short peptides can mimic the ligands of lectin receptors, including siglecs, for this purpose. Peptide mimetics of sugars have potential advantages over glycans and glycoproteins because of the ease of chemical synthesis, their flexibility in design, and favorable physical properties. Multivalent peptides can be constructed that have much higher avidities to lectins than monosaccharides and are similar in binding avidity to natural multivalent glycoproteins and glycoconjugates such as fetuin and mucin [29,30]. A number of peptides that mimic sugars have been identified, some of which closely resemble specific sugars and bind to oligosaccharide-binding antibodies [31–36]. Some peptides can functionally mimic a sugar, such as those with the consensus core sequence YPY that inhibit the mitogenic activity of the Man-specific lectin concanavalin A, yet bind at a site different from the saccharide-binding site [37,38]. Peptide mimetics of carbohydrate antigens have been studied as vaccines to elicit antibodies against sugar antigens, including those on the surface of HIV, and complex oligosaccharides [36,39].

We initially identified several sequences of amino acids that bound to specific plant lectins by screening phage display libraries [40]. Sequences were further refined by *in silico* modeling of docking of a peptide into the sugar binding site of the crystal structures of lectins. These studies predicted that several short peptide sequences, 5 to 8 amino acids in length, would have significant affinity to lectins chosen as analogs of cell-surface receptors. The sequences identified by this approach were incorporated into tetravalent structures, a design based on the concept of avidity as a function of ligand density and entropic factors [41–43] and also to accommodate the possibility that cross-linking of receptors may be required for modulation of signal transduction pathways [44,45]. In binding assays, the peptide with the sequence HPSLK (designated sv6B) had characteristics of a general sugar mimetic and bound to several lectins that bind monosaccharides such as Neu5Ac, Gal, GalNAc or fucose with avidities several orders of magnitude greater than their natural ligands [29,30]. Surprisingly, when the HPSL core was retained but the peptide lengthened to NPSHPSLG (designated svH1D), and in particular the SL→LS inversion to generate NPSHPLSG (designated svH1C), the peptides did not bind to lectins that bound monosaccharides but bound strongly to those specific for di- or tri-saccharides that terminate with Neu5Ac-Gal. When svH1C was added to cultures of human peripheral blood mononuclear cells (PBMCs), endocytosis of opsonized microspheres by adherent cells was activated [29,30]. Additionally, svH1C stimulated phosphorylation of STAT6, an indication that the peptide triggered a receptor-mediated signal transduction pathway [29]. These observations suggested that the peptide has biological activity and may bind productively to specific receptors. As a step to further understand the mechanism of action of svH1C, we hypothesized that, as a mimetic of Neu5Ac-Gal, the peptide binds to receptors such as siglecs and NKG2D that are specific for these sugars.

The amino acid residues in the binding sites of many lectins and receptors have been identified and, although the protein structures obtained from data bases are rigid, the validity of the *in silico* approach to predictions of binding energies for peptides is supported by the concept that flexible ligands select specific conformations of a binding site according to the Monod-Wyman-Changeux hypothesis [46]. To confirm that the peptide interacted with a glycan binding site from a flexible, non-structured conformation, CD and NMR spectra of svH1C were examined. Although an unusual CD signal was observed at elevated concentrations, a specific structure for the peptide was not observed by NMR spectroscopy. We designed solid-phase assays in which binding of biotin-tagged svH1C to recombinant receptors occurred under conditions in which the peptide sequences retained full flexibility. We also observed binding of the peptide to a subset of the monocyte population in human PBMCs after treatment of cells with neuraminidase. Furthermore, svH1C induced activation and proliferation of immune cells in the peritoneal cavity of mice.

## Materials and Methods

### Ethics Statement

Studies of peritoneal cells were performed by Biomodels, LLC, Watertown, MA, which is accredited by the Association for Assessment and Accreditation of Laboratory Animal Care International. The protocol was approved by Biomodels’ IACUC and has IACUC approval number 13-0611-01. C57BL/6 mice were obtained from Charles River Laboratories (Wilmington, MA). The studies were performed in animal rooms provided with HEPA filtered air at a temperature of 70°F +/-5°F and 50% +/-20% relative humidity. Animal rooms were set to maintain a minimum of 12 to 15 air changes per hour. The room was on an automatic timer for a light/dark cycle of 12 hours on and 12 hours off with no twilight. A cage card or label with the appropriate information necessary to identify the study, dose, animal number, and treatment group marked all cages. Animals were identified by an ear punch corresponding to an individual number. Animals were fed with a sterile Purina Labdiet 5053 rodent diet and water was provided ad libitum.

Human peripheral blood mononuclear cells (PBMCs) were prepared from purchased discarded cellular residues of a plasma preparation procedure (TRIMA) performed on blood of anonymous donors at the Blood Centers of the Pacific, San Francisco, CA, and sold commercially for research use.

### Synthesis of svH1C

The amino acid sequence identified previously [29,30] as a Neu5Ac-Gal- mimetic was incorporated into a tetravalent peptide that has the structure [(NPSHPLSGGGGS)_2_K]_2_K-NH_2_. The tetravalent peptide was synthesized by CBL Biopharma (Patras, Greece) on a tri-lysine core by standard procedures [47] utilizing Fmoc (9-fluorenylmethoxycarbonyl)-protected amino acids. The sequence–GGGS- is a linker that extends the mimetic from the tri-lysine core. Modifications at the C-terminus made during synthesis consisted of an amide group (no tag) or an extension of the peptide by addition of ε-biotinyl-lysine-amide for the binding assays. The ‘arms’ were synthesized separately by solid-phase synthesis by standard chemistry and subsequently condensed in solution with the four amino groups generated by the tri-lysine core [48]. Quality of the synthetic product was assessed by HPLC and mass spectroscopy. Purity of the peptide was 97.7%, with net peptide content 73% without counter-ion and 87% when the counter-ion was included. The remaining material was largely water of hydration of the polar peptide. Solutions of the peptide in water were neutralized and passed through a 1 x 10 cm column of DEAE-Sephadex A-25, Cl^-^ form, at pH 5 to 6 to remove trifluoroacetic acid.

For *in vivo* studies, svH1C was prepared by absorption of 1 g peptide on a 2.5 X 10 cm column of CM-Sephadex C-25 in 100 mM NaCl. The column was washed with 200 mM NaCl and svH1C was then eluted with 500 mM NaCl that was prepared in endotoxin-free water (<0.005 EU/mL, HyClone Laboratories, Logan, UT). Endotoxin was undetectable (<0.01 EU/10 mg peptide) in the eluted peptide as assayed by a quantitative colorimetric LAL assay (Lonza, Walkersville, MD). Peptide concentration was determined by the bicinchoninic acid assay (Pierce Biotechnology, Rockland, IL) using a dansylated peptide (extinction coefficient, ε_mM_ = 5.7 cm^-1^ at 336 nm) as standard or with a calculated [49,50] svH1C extinction coefficient of 25 OD_210 nm_/mg/mL.

### CD and NMR

CD spectra were obtained with 100 μM svH1C dissolved in 50 mM sodium borate, pH 9.0, using a JASCO J-170 CD spectropolarimeter operated in continuous scan mode and 200 nm/min scan speed. Spectra were generated from five serial accumulations. Samples were either diluted with water or adjusted to pH 2 for additional spectra.

Samples of the tetravalent peptide in H_2_O/D_2_O (90%/10% v/v) were analyzed by NMR at 298K with a Bruker Avance DPX 400 MHz spectrometer. ^1^H 1D NMR spectra were recorded using spectral width of 12–17 ppm with presaturation of the H_2_O signal. ^1^H-^1^H 2D TOCSY [51,52] were recorded using the MLEV-17 spin lock sequence using τ_m_ = 80 ms, and ^1^H-^1^H TPPI NOESY [53,54] spectra were acquired using mixing time τ_m_ = 250 or 300 ms applying water suppression during the relaxation delay and mixing time. All 2D spectra were acquired with 16.0204 ppm spectral width, consisting of 2K data points in the F2 dimension, 64 transients and 512–1024 complex increments in the F1 dimension. Raw data were multiplied in both dimensions by a pure cosine-squared bell window function and Fourier-transformed to obtain 2048×2048 real data points. A polynomial base-line correction was applied in both directions. For data processing and spectral analysis, the standard Bruker software (Topspin 1.3) and XEASY program [55] (ETH, Zurich) were used.

### Binding assays

Isothermal microcalorimetry was performed by TA Instruments Professional Services (New Castle, DE) by titrating 10 μM NKG2D (monomer concentration) (R&D Systems, Inc., Minneapolis, MN) with 100 μM svH1C in 50 mM Tris-HCl, pH 7.5, 150 mM NaCl, 0.1 mM EDTA. Recombinant human receptors were obtained from R&D Systems as chimeric proteins. NKG2D is a type II transmembrane protein and the extracellular lectin-type domain was fused at the N-terminus with a human IgG_1_ Fc domain. Recombinant siglecs contained a C-terminal IgG_1_ Fc domain or a His_6–10_ tag. The lyophilized proteins were reconstituted in phosphate-buffered saline, pH 7.2 (PBS), to 100 μg/mL. The Fc-chimeric proteins were bound to protein A/G-coated wells of microtiter plates (Pierce Biotechnology). His-tagged proteins were bound in Nickel-coated plates (Pierce Biotechnology). Sufficient proteins were added to wells to saturate the binding capacity of the wells. After a wash to remove unbound receptor, wells were blocked with 1% gelatin in wash buffer. Wash buffers were either 10 mM phosphate, pH 7.2; 10 mM HEPES, pH 7.4; or 50 mM Tris-HCl, pH.7.5, each in 150 mM NaCl containing 0.05% Tween-20. Biotinylated peptides were prepared as 2 μM solutions in the wash buffer and 50 μL (100 pmoles) were added to each well and incubated at room temperature for one hour. After four washes with 350 μL buffer containing 0.05% Tween-20, each assay was incubated with 50 μL of 1 μg/mL streptavidin-peroxidase (Sigma-Aldrich, St. Louis, MO). The wells were again washed four times with buffer-Tween-20 and then 50 μL peroxidase substrate (1-Step ULTRA TMB, Pierce Biotechnology) were added. The reaction was allowed to proceed at room temperature for 5 min, and the reaction was stopped with 50 μL of 2 N H_2_SO_4_ or 1 M H_3_PO_4_. Absorbance was measured at 450 nm and converted to the amount of streptavidin-peroxidase bound with the specific activity of the conjugate determined under the conditions of the assay. Absorbance of assays with svH1C was corrected for background levels of binding of streptavidin to the receptors in wells without a peptide or with an inactive peptide, which were less than 10% of the value with bound peptide, except for Siglec-5 and Siglec-14. Binding of streptavidin to these siglecs caused a background level of nearly 50% of binding in the presence of svH1C. Interestingly, the sequences of the first 200 amino acids of these two siglecs are identical [18], which suggests that streptavidin bound directly to these receptors. For inhibition studies, fetal bovine fetuin (purity, 88%; cat. no. 341506, Calbiochem, EMD Chemicals, Philadelphia, PA) and sialyllactose (cat. no. A0828, Sigma-Aldrich) were added to the assay along with svH1C.

Molecular modeling was performed of docking of a single (monovalent) sequence into the glycan-binding site of cell-surface receptors. The crystal structures of the receptors were down-loaded from the Protein Data Bank into the docking mode of ArgusLab 4.0.1 software (Mark A Thompson, Planaria Software LLC, Seattle, WA, http://www.arguslab.com), which was used for modeling. Amino acid residues that comprise the binding site of a receptor were selected from the literature that describes each protein.

### Binding of biotin-tagged svH1C to cells

Human PBMCs in RPMI-1640 medium containing 10% fetal bovine serum (FBS) were washed two times with RPMI-1640 medium without FBS. Cells (1 x 10^6^ in 100 μL medium) were digested with 10 mU α(2→3,6,8,9)neuraminidase from *Arthrobacter ureafaciens* (Sigma-Aldrich) for 60 min at 37°C, washed two times with cold PBS and then incubated 30 min on ice with 1 μM biotin-tagged svH1C in PBS. The cells were washed two times with PBS and stained 20 min on ice with CD3-APC, CD4-PE-Cy7, CD14-APC-Cy7, CD16-FITC, CD56-PE, plus streptavidin-PerCP-Cy5.5, washed two times with PBS and fixed with 1% formaldehyde for flow cytometry.

### Phospho-immunoreceptor assay

Human PBMCs were incubated at a density of 2 x 10^6^ cells/mL overnight in RPMI-1640 medium supplemented with 0.1% ovalbumin, 1:100 dilution of penicillin-streptomycin solution (Mediatech, Inc., Herndon, VA), and 1 mM glutamine at 37°C and 5% CO_2_. svH1C was added to a final concentration of 100 nM, and after 5 min the cells were collected by centrifugation at 500g for 2 min. The cells were lysed and spread on an array of capture antibodies against cell-surface receptors on immune cells (Human Phospho-Immunoreceptor Array, R&D Systems). The protocol for detection of phosphorylated receptors with an anti-phospho-tyrosine antibody conjugated to horseradish peroxidase and detection by chemiluminescence with x-ray film was followed as described by the manufacturer. Spots on the film were analyzed by ImageJ software.

### *In vivo* studies

C57Bl/6 mice, 22–24 g body weight, were injected subcutaneously with 1 nmole svH1C per g on days 0, 2 and 4. On days 1, 3 and 5, primary peritoneal cells from three animals were isolated and pooled, and the populations of macrophages, dendritic cells (DCs), CD3^+^, CD4^+^ and CD8^+^ T cells, natural killer (NK) cells and B cells were evaluated by flow cytometry with specific biomarkers. FACS was performed using a MACSQuant Analyzer (Miltenyi Biotec, Teterow, Germany). Data were analyzed with FlowJo software, version 10.0.6.

## Results

### Insight into the structure of svH1C

**Fig 1:**
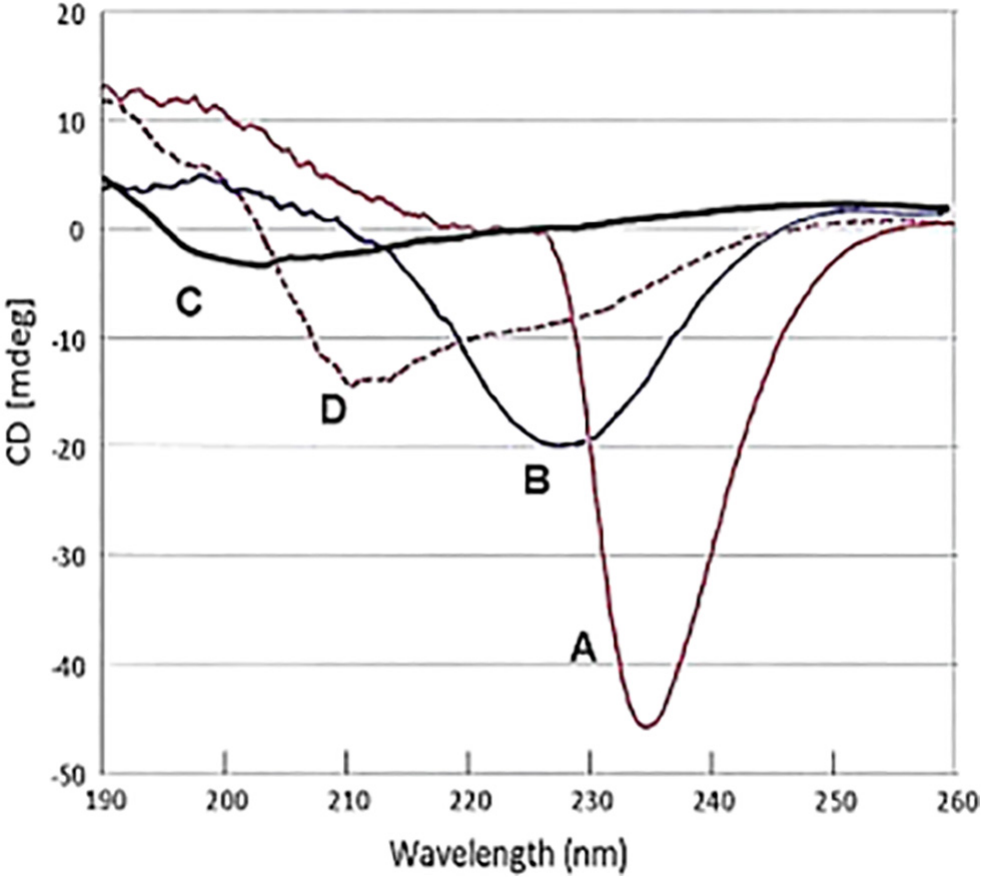
Circular dichroism spectra of svH1C as a function of concentration. (A) 100 μM peptide in 50 mM borate, pH 9.0; (B) peptide solution in (A) diluted 1:3 with water; (C) peptide solution in (A) diluted 1:9 with water; (D) 100 μM peptide in 50 mM borate adjusted to pH 2.

As an initial approach to structural analysis, CD spectra were performed with svH1C in borate buffer, pH 9. The spectrum for a 100 μM solution of the peptide had a strong negative signal at 234 nm (Fig 1, curve A). The minimum broadened and shifted to 227 nm when diluted to 25 μM (Fig 1, curve B), and disappeared when the peptide was diluted to 10 μM, with a new minimum at 203 (Fig 1, curve C). The unusual spectra at higher concentrations may be a function of the tetravalent structure of the peptide, because similar negative peaks were observed between 225 nm and 235 nm with 100 μM solutions of several other tetravalent peptides with similarly short but different sequences. When the sample that provided spectrum A in Fig 1 was acidified to pH 2, the minimum at 234 nm was replaced with a minimum at 211 nm and a shoulder at about 230 nm (Fig 1, curve D). This spectrum suggests a weak α-helical structure [56], but formation of helical structures is restrained by the two proline residues in each arm. These spectra suggest that the peptide aggregates at the higher concentrations. However, because the receptor binding studies were performed with 2 μM peptide, svH1C was likely in a random, flexible structure under those conditions.

**Fig 2:**
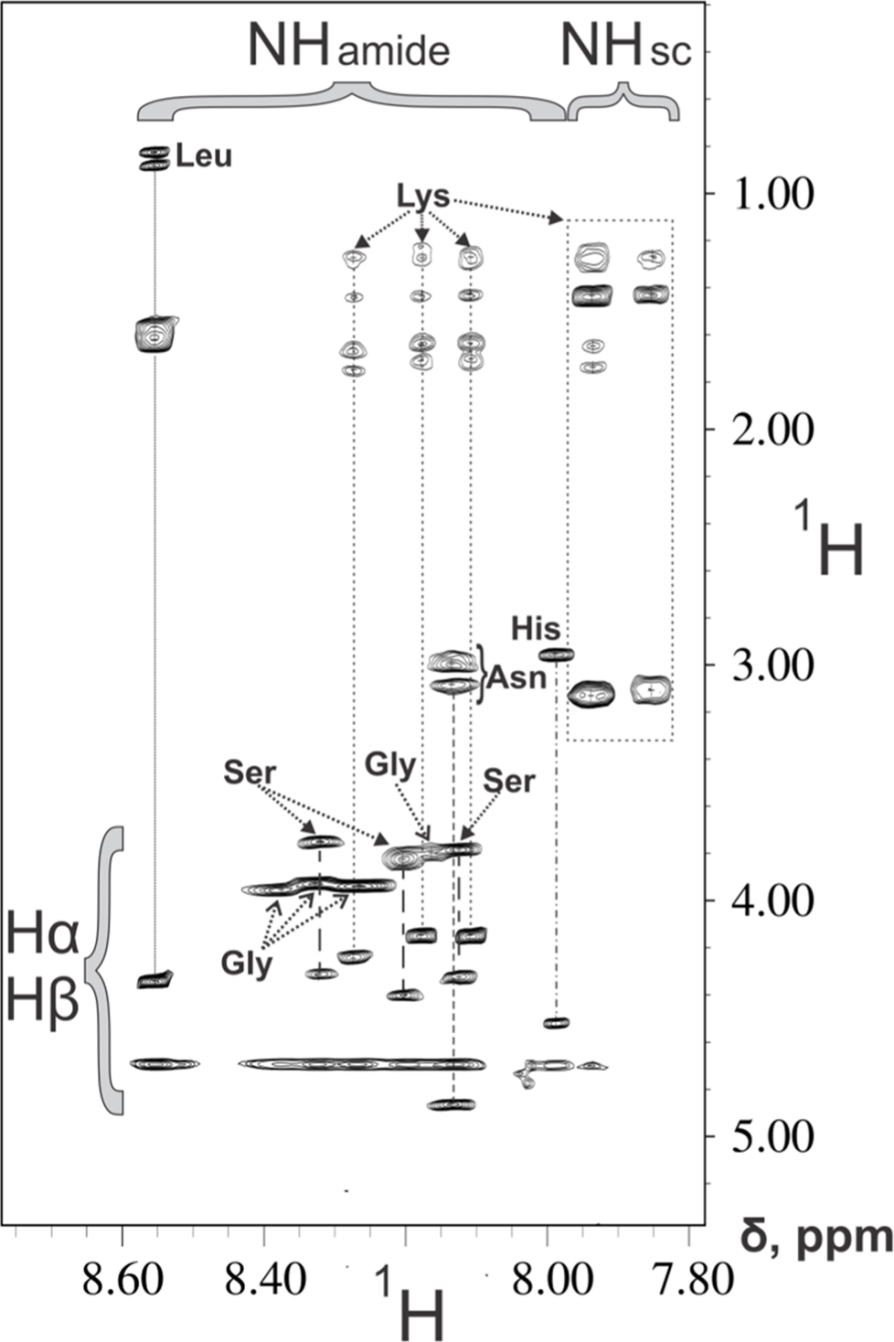
^1^H-^1^H 2D TOCSY 400 MHz NMR spectrum of svH1C. The fingerprint of HN-Hα/aliph svH1C protons on a 16.0204 ppm spectrum was recorded in H2O/D2O (90%/10% v/v, at pH 7.0, T = 298K). The amino acid spin-patterns are indicated with dashed lines. Vertical bracket indicate the HN-Hα & ΗΝ-Ηβ (only for serine residues), while horizontal brackets denotes the amide HN and the lysine side-chain NH groups, which are involved in iso-peptide bonds.

NMR spectra were determined with solutions of 1 mM svH1C in PBS (S1 Fig). Attempts to perform sequence specific assignment for the peptide faced the drawback of resonance degeneration and lack of a defined tertiary conformation, indicative of flexibility of the svH1C arms. Analysis of the TOCSY spectra was based on the hypothesis that the identical residues existing in each unit of the tetramer resonate at the same (or extremely similar) field. Consequently, the individual spin patterns were identified according to (i) the intra-residue connectivities, and (ii) the characteristic spin-patterns of each of the amino acids present in the peptide sequence. A single set of peaks was assigned to each of the residues of the svH1C segment/unit: [NPSHPLSGGGGS]K. Because of the different chemical structures of the three lysine residues, which are linked not only through the typical peptide bonds but also through iso-peptide bonds (connectivities between the iso-peptide HN groups and the side-chain protons were identified for two of the three lysine residues), we managed to attribute three spin-patterns to the three lysine residues of the svH1C peptide (S1 Table). Standard sequence specific proton assignment was also prevented by the fact that NOESY spectra (250–300 ms mixing time) lacked any meaningful inter-residue connectivity (*i*.*e*., Hα(i)-HN(i+1)). Data from the ΗΝ regions of the 2D ^1^H-^1^H TOCSY spectra are shown in Fig 2 (the ^1^H 1D spectrum is shown in S1 Fig), which suggest the absence of a well-ordered structure. These analyses indicate that binding to lectin-type receptors occurred with flexible peptide arms capable of forming conformations that accommodate glycan binding sites.

### Binding of svH1C to siglecs

Tetravalent svH1C, with the mimetic sequence NPSHPLSG, bound selectively to lectins from *Sambucus nigra* (SNA1) and *Maackia amurensis* (MAA) [29,30]. MAA is a mixture of *M*. *amurensis* leukoagglutinin (MAL) and *M*. *amurensis* hemagglutinin (MAH), both of which along with SNA1 bind preferentially to terminal Neu5Ac-Gal- sequences on complex glycans [57]. Binding of the peptide to several lectins specific for monosaccharides was not detected [30]. We then assayed binding of svH1C to several siglecs, which comprise a family of cell-surface receptors that are specific for Neu5Ac-Gal/GalNAc- sequences. Within this family of receptors are proteins that bind to Neu5Ac(α2,3 or α2,6)Gal, Neu5Ac(α2,6)GalNAc or Neu5Ac(α2,8)Neu5Ac [8–12]. For binding assays, sufficient recombinant homodimeric, chimeric proteins containing a siglec domain and a Fc domain per subunit were added to protein A/G-coated microtiter wells to saturate the binding sites. Recombinant Siglec-1 contained a C-terminal His tag rather than a Fc domain and was bound in Nickel-coated wells. Non-specific sites in the wells were blocked with 1% gelatin-0.05% Tween-20 in the wash buffer.

**Fig 3:**
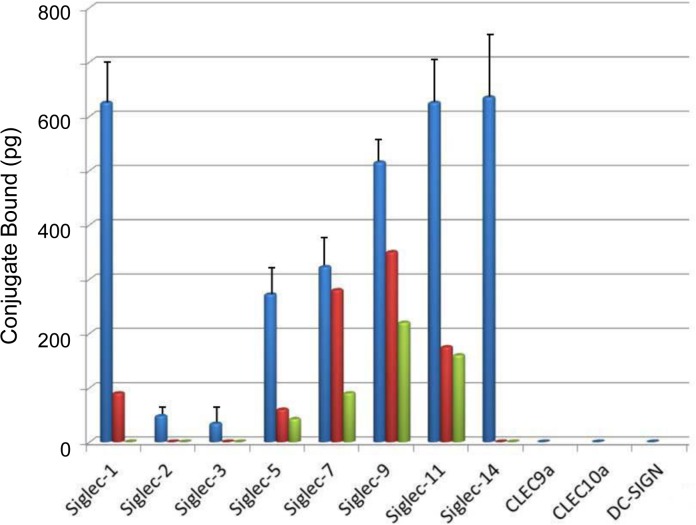
Binding of svH1C to lectin-type receptors. The buffer in these assays was PBS containing 0.05% Tween-20 (see text for effects of different buffer compositions). The figure shows the amount of streptavidin-peroxidase bound to svH1c that was bound to the receptors. Siglec-1 and CLEC10a contained a C-terminal His tag and were assayed in separate experiments. The other receptors were Fc-chimeras and were included in the same assays. SEM was determined for six assays from four independent experiments. Inhibition by fetuin is shown by the average of single values in two assays in which the glycoprotein was added at 10 μM (red) or 30 μM (green).

Extent of binding of biotinylated svH1C to siglecs was assayed with three different buffers, either 10 mM phosphate, pH 7.2; 10 mM HEPES, pH 7.4; or 50 mM Tris-HCl, pH 7.5, each in 150 mM NaCl-0.05% Tween-20 (see **Materials and Methods**for details). Binding data in phosphate-NaCl (PBS) are presented in Fig 3. Strong binding was found except for Siglec-2 and Siglec-3, which are specific for Neu5Ac(α2,6)Gal. Among the siglecs assayed, greatest binding occurred consistently with Siglec-1, which prefers Neu5Ac(α2,3)Gal, a specificity shared with Siglec-9 and NKG2D. Strong binding was found with Siglec-5 and Siglec-14, which are specific for Neu5Ac(α2,8)Neu5Ac and/or Neu5Ac(α2,6)GalNAc [9–12], and apparently function as paired receptors on monocytes [12,18]. Siglec-7 and -11 also have a preference for binding Neu5Ac(α2,8)Neu5Ac.

The composition of the buffer had a significant effect on binding of peptide to individual siglecs. Interestingly, Siglec-2 and Siglec-3 bound svH1C as strongly as the other siglecs in assays with Tris buffer. In contrast, the most discrimination of binding between the various siglecs was observed when HEPES buffer was used, with little or no binding detected to Siglec-2 or Siglec-3, which was similar to results in PBS (Fig 3). Binding of svH1C to Siglec-7, -9, and -11 was significantly less with HEPES buffer as compared with PBS. These results suggest that the peptide binds to all the siglecs but with differing avidities, with only the strongest interactions surviving the extensive washes. Binding of the peptide was inhibited by the Neu5Ac-rich, multivalent glycoprotein, fetuin.

Consistent with results from binding of peptides to lectins [29,30], biotinylated tetravalent peptides with the sequence HPSLK (sv6B) and NPSHPSLG (svH1D) also bound to siglecs, although with avidity patterns different from that of svH1C. In contrast, no binding occurred with a peptide with the structure [(VGGGSGGGS)_2_K]_2_K-ε-biotinyl-K-NH_2_, which was used as a negative control. No binding was detected with the lectin receptors CLEC9a (ligand unknown), CLEC10a (specific for GalNAc) or DC-SIGN (specific for Man). Binding of svH1C to these receptors was not detected regardless of the buffer used. Addition of CaCl_2_ and MgCl_2_ (1 mM each) did not increase binding of svH1C.

### Binding of svH1C to NKG2D

**Fig 4:**
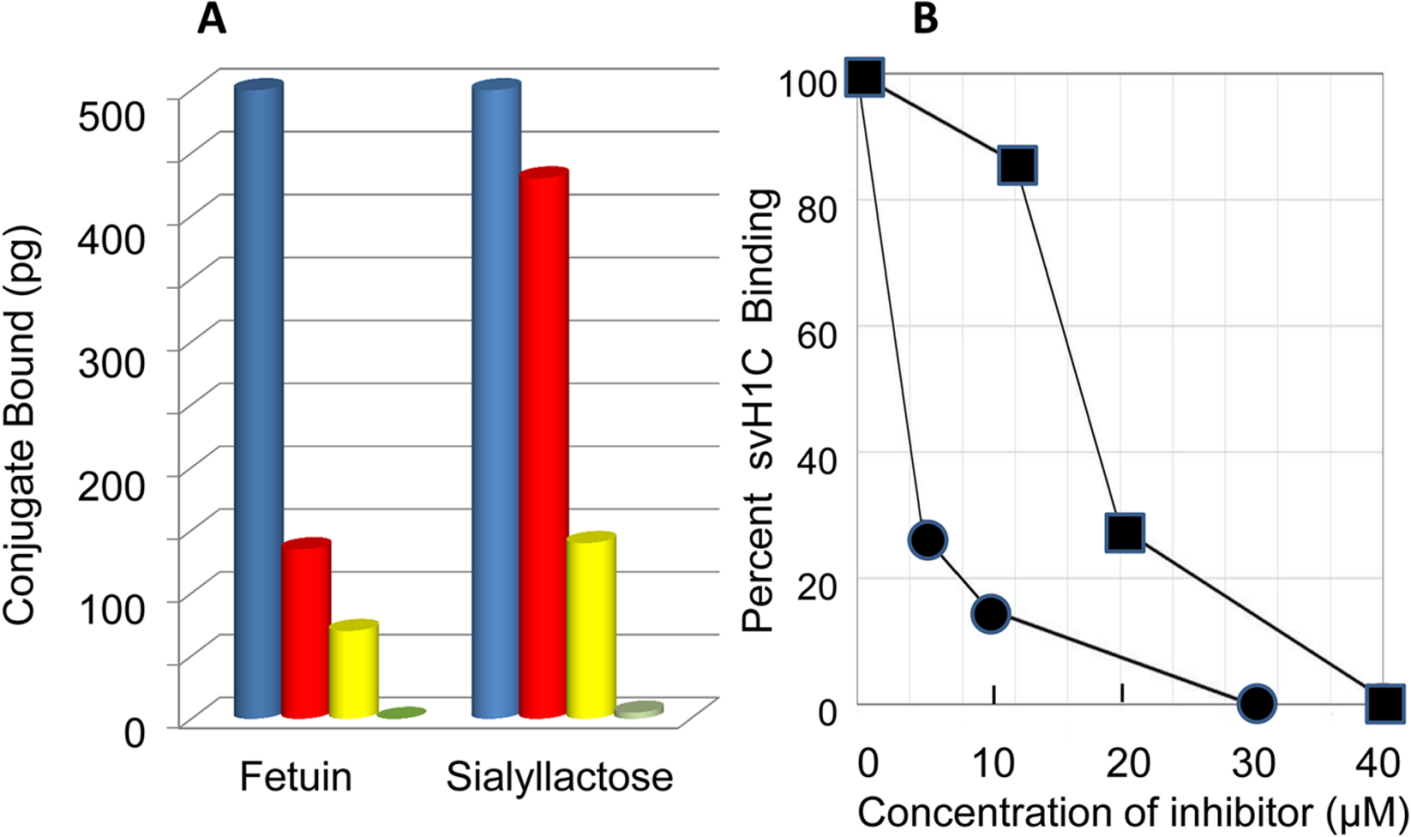
Binding of svH1C to NKG2D. (A) The amount of bound peptide was measured after extensive washing with PBS containing 0.05% Tween-20. Fetuin (5 μM, red; 10 μM, yellow; 30 μM, green) and sialyllactose (12 μM, red; 20 μM, yellow; 40 μM, green) were included as inhibitors. Inhibition is shown by the average of single values from three separate assays. The average 100% value was 2.4 ng conjugate bound. (B) Graphical representation of inhibition of binding of svH1C to NKG2D by fetuin (circles) or sialyllactose (squares) as shown in (A).

NKG2D is a homodimeric, type II receptor whose recognized ligands are cell-surface proteins MICA and MICB, which are major histocompatibility complex class I homologs. Other polypeptide ligands include ULBP, Rae-1 and H60 [26]. Extensive studies of binding of MICA and MICB [24–26] have shown that each homodimer binds a single ligand at the interface of the subunits. Each subunit of NKG2D also contains a lectin domain that binds glycans with specificity for Neu5Ac(α2,3)Gal [27]. With the assay as used for siglecs, we found that svH1C bound strongly to NKG2D. Binding was inhibited by fetuin (Fig 4A), with an IC_50_ of approximately 3 μM protein, when added with 2 μM svH1C (Fig 4B). Fetuin was as effective an inhibitor of binding of svH1C to NKG2D as it was of the binding of the peptide to siglecs. Fetuin contains three N-linked and three O-linked glycans per molecule [58–62], with trisialylated or tetrasialylated termini that are linked nearly equally as Neu5Ac(α2,3)Gal or Neu5Ac(α2,6)Gal. We previously found fetuin effectively competed with svH1C for binding to plant lectins, and that digestion of fetuin with neuraminidase nearly eliminated its ability to inhibit binding of svH1C [29]. From the number of multivalent glycans on each fetuin molecule, we concluded that svH1C effectively competed with natural multivalent glycan ligands for the same binding site on these receptors.

Inhibition of binding of svH1C to NKG2D by sialyllactose further supported binding of the peptide in the glycan-binding site. Sialyllactose was a relatively weak inhibitor (IC_50_ ≈ 18 μM), which may have resulted because the Neu5Ac(α2,3)Gal linkage, which is the preferred ligand for NKG2D, was a minor component of the mixture of sialyllactose isomers, among which the isomer with the Neu5Ac(α2,6)Gal linkage was the major form (Fig 4A and 4B).

We then assayed binding of svH1C to NKG2D directly by isothermal microcalorimetry. svH1C (100 μM) was titrated into a solution of NKG2D (10 μM, subunit concentration) and changes in heat content of the system were measured. The concentration of NKG2D was introduced into the data analysis as the total number of lectin-like binding sites. The titration curve (S2 Fig) yielded a K_A_ = 1.7 x 10^6^ M^-1^ (K_D_ = 0.58 μM). The dissociation constant corresponds to a ΔG’ = -38.4 kJ/mol. The ΔH of the binding reaction determined by microcalorimetry was positive, 16.4 kJ/mol, which suggested that entropy was a major factor in the energy of binding. The affinity of svH1C for NKG2D was similar to that of the endogenous polypeptide ligands, which have K_D_ values in the range of 0.6 to 1.0 μM [24,25].

In the analysis of the microcalorimetry data, the stoichiometry, *n*, yielded a value of 0.71, which suggests that each binding site on approximately half of the dimeric protein was filled with one peptide molecule while the binding sites on the remaining dimers were either cross-linked by one peptide or that only one of the subunits was bound with a peptide. If each arm of the tetravalent structure was in the extended β-conformation, as the CD and NMR data in Figs 1 and 2 suggest, the distance from each mimetic domain to another is approximately 70Å. This distance is sufficient to span the approximately 34Å between the lectin domains in the structure of the dimeric NKG2D [24]. Thus it is feasible that the two binding sites on the dimeric protein are accessible to a single peptide molecule. As an alternate interpretation of the stoichiometry, a fraction of the receptor may not have achieved the native conformation during reconstitution.

**Fig 5:**
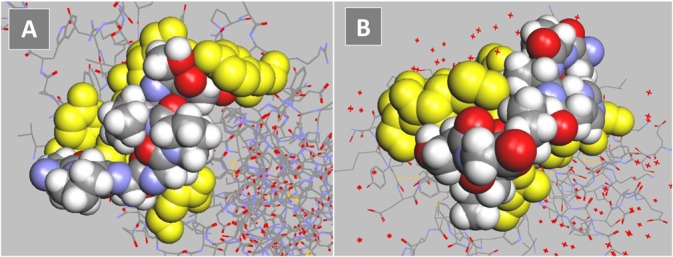
Models of monovalent, mimetic sequence of svH1C (colored space-filled structure) docked in the ligand binding site of receptors (yellow). (A) Siglec-5 (accession no. 2ZG1), predicted binding energy, -47 kJ/mol. (B) NKG2D (accession no. 1MPU), predicted binding energy, -40 kJ/mol.

The inhibition by fetuin of binding to siglecs and NKG2D indicated that the dissociation constants for svH1C were similar with the two types of receptors. Molecular modeling was used to compare the predicted energies of binding of the peptide to Siglec-5 and NKG2D. The glycan binding site for Siglec-5 was described by Zhuravleva et al. [23]. The predicted binding energy calculated by the docking software was -47 kJ/mol (Fig 5A). A model of NKG2D, with the binding site as described by Li et al. [24] and McFarland et al. [25], provided a predicted binding energy of -40 kJ/mol (Fig 5B). This value is similar to the binding energy calculated from the dissociation constant determined by microcalorimetry.

### Binding of svH1C to cells

**Fig 6:**
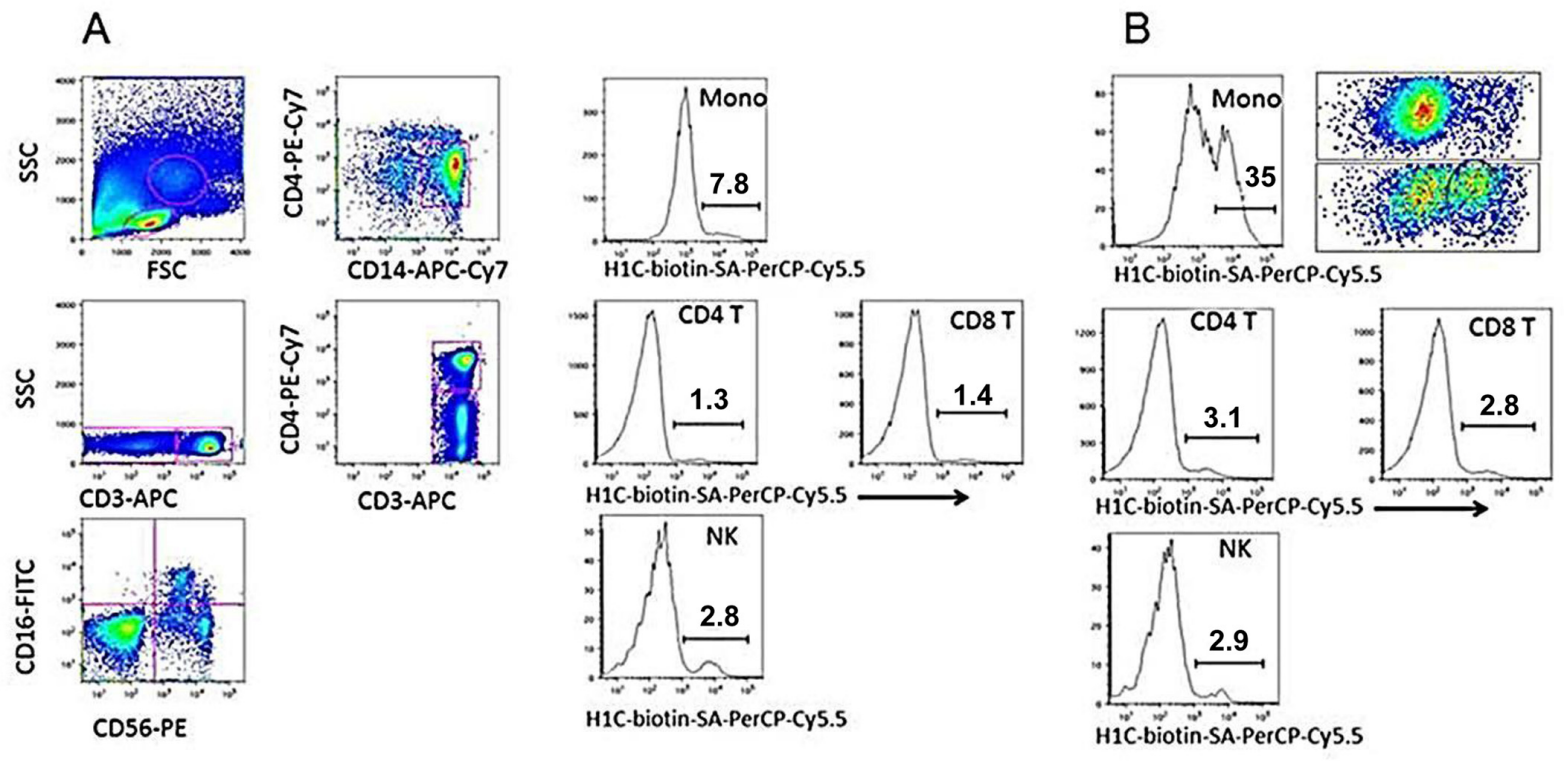
svH1C bound to monocytes after digestion of PBMCs with neuraminidase. (A) Dot plots and histograms of cells in PBMC samples treated 30 min at 37°C with neuraminidase inactivated by heating 20 min in boiling water. (B) Histograms of PBMCs from the same donor as (A) after treatment with active neuraminidase. After enzyme digestion, cells were incubated 30 min on ice with 1 μM biotin-tagged svH1C, washed and incubated 30 min on ice with marker antibodies and streptavidin labeled with PerCP-Cy5.5. Binding to only the monocyte (mono) fraction was significantly increased by treatment with neuraminidase, as shown by the histogram. The upper dot plot in (B) represents the monocyte fraction from (A), whereas the lower dot plot represents the monocyte fraction from (B). The subset of monocytes to which svH1C bound is circled, which accounted for 35% of the total monocyte population. This experiment was performed three times with similar results.

The siglecs to which svH1C bound most strongly (Fig 3) are expressed on monocytes [9–12]. To determine whether binding of svH1C to receptors could be observed with whole cells, human PBMCs were incubated on ice with biotinylated svH1C in PBS for 30 min, washed with PBS, stained with labeled antibodies against cell-surface markers plus streptavidin-PerCP-Cy5.5, washed and fixed. By flow cytometry, streptavidin was detected on small subsets of NK, CD4^+^ and CD8^+^ T cells. However, no binding was detected with monocytes. Collins et al. [63] and Zhang and Varki [64] found that binding of multivalent Neu5Ac-containing glycans to B cells was detected only after digestion of the cells with neuraminidase to remove cell-surface Neu5Ac. We repeated the experiment to include a treatment of PBMCs with neuraminidase at 37°C before cooling the cells on ice and incubating with biotin-tagged svH1V on ice. The binding step was performed on ice to prevent endocytosis of the peptide that could be mediated by siglecs [11,12]. As shown in Fig 6B, significant binding was found with a subpopulation among CD14^+^ monocytes after cells were treated with neuraminidase. Treatment with neuraminidase did not have a significant effect on binding of svH1C to NK, CD4^+^ or CD8^+^ T cells. Histograms of samples treated with neuraminidase that was inactivated by heating in boiling water (Fig 6A) were similar to the control without peptide.

### Effect of svH1C on receptor phosphorylation

We then asked whether svH1C would alter the phosphorylation status of cell-surface receptors. Human PBMCs were cultured overnight in RPMI-1640 medium supplemented with 0.1% ovalbumin, which was added in place of serum to minimize effects of possible regulatory factors in serum. svH1C was added to 100 nM and cells were collected after 5 min of further incubation. Cells were lysed and spread on an array of cell-surface receptor antibodies (see **Materials and Methods**). After washes, an anti-phospho-tyrosine antibody conjugated to horseradish peroxidase was added and phosphorylated tyrosine was detected by chemiluminescence.

**Fig 7:**
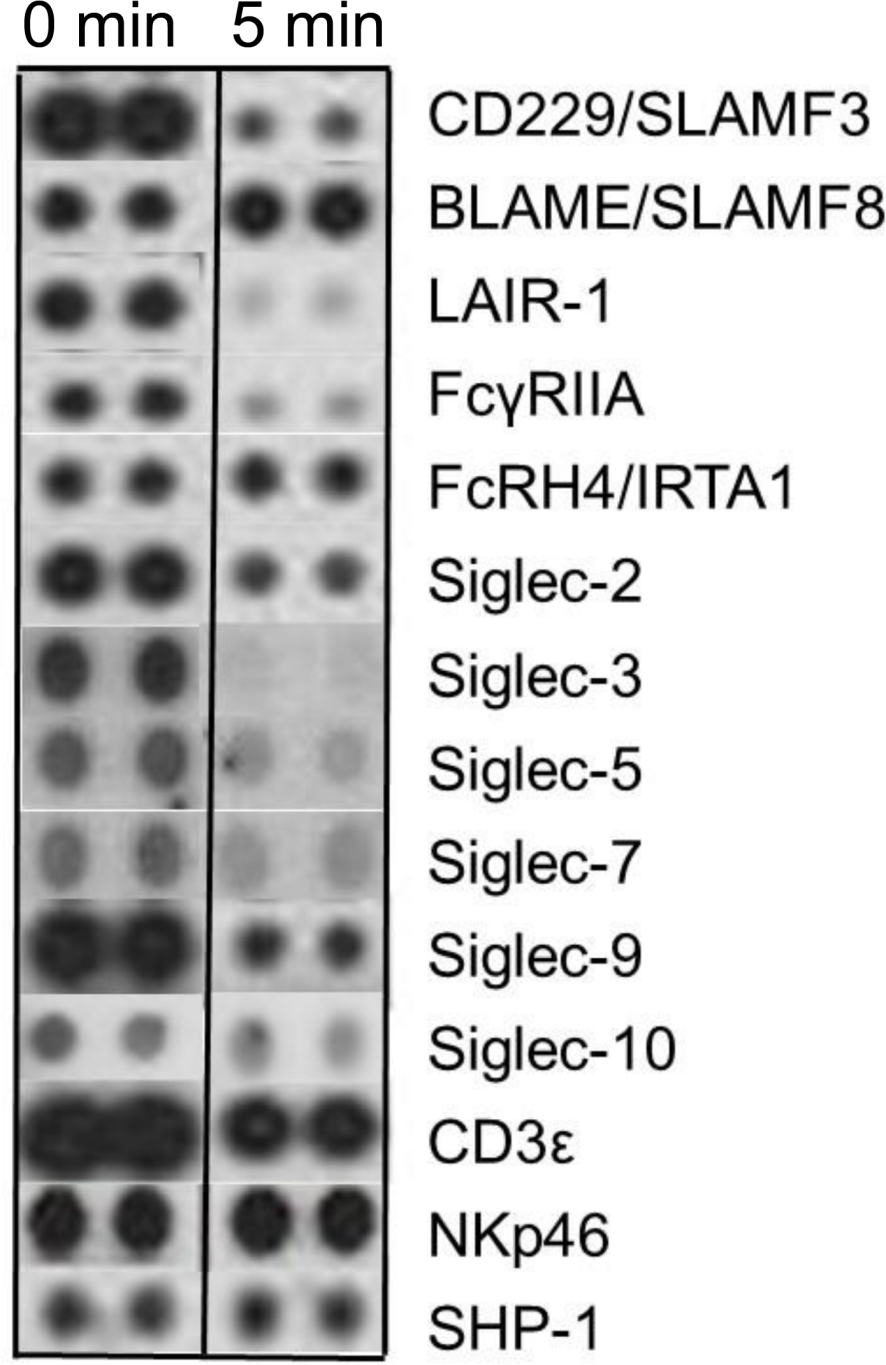
Phosphorylated cell-surface receptors after treatment of human PBMCs with 100 nM svH1C for 5 min. The cells were collected by centrifugation, lysed and analyzed by the Human Phospho-Immunoreceptor Array (R&D Systems) according to the manufacture’s protocol. This experiment was performed in duplicate two times with similar results.

Sections of films corresponding to a selection of receptors are shown in Fig 7. Phosphorylated siglecs included on the array were decreased by more than half, with the greatest reduction on Siglecs-3, -5 and -7 in cells treated with svH1C. The amounts of phosphorylated CD229/SLAMF3, LAIR-1 and FcγRIIA were also dramatically reduced. However, phosphorylation of FcRH4/IRTA1, NKp46 and SHP-1 was not changed, while that of BLAME/SLAMF8 increased. The major phosphorylated protein, the ε subunit of the CD3 T cell receptor complex, was slightly reduced. These effects indicated that the peptide interacted with cells and, by inducing dephosphorylation, attenuated the activity of inhibitory receptors.

### Effect of svH1C on immune cells *in vivo*

**Fig 8:**
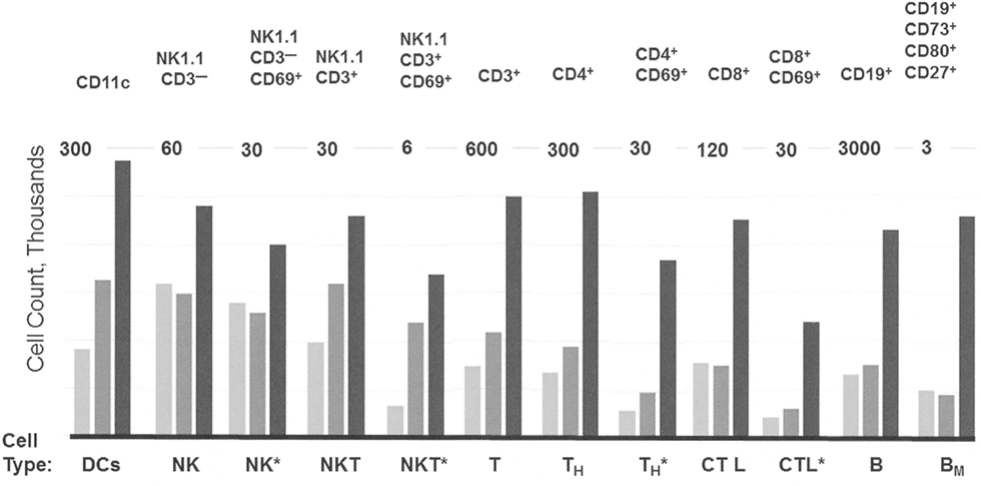
Increases in populations of immune cells in the peritoneal cavity. svH1C was injected subcutaneously every other day with a dose of 1 nmole/g into C57Bl/6 mice. Peritoneal cells were obtained from 3 animals, pooled, and analyzed by flow cytometry. The bars, in increasing darkness, show populations of specific cell types at day 1, 3 and 5 of treatment, i.e., 24 hours after each injection at day 0, 2 and 4. The markers used to identify cell types are listed across the top of the figure. The total number of each cell type was plotted, with the scale indicated at the top of each cell type. Cells are identified by the usual designations across the bottom of the figure. B_M_ indicates memory B cells. An asterisk indicates activated populations that expressed CD69.

To determine whether a decrease in activity of inhibitory receptors is reflected by proliferation of immune cells *in vivo*, svH1C was injected subcutaneously every other day into C57Bl/6 mice at a dose of 1 nmole per g body weight. Injections were administered on day 0, 2 and 4, and peritoneal lavage was performed to obtain primary cells 24 h after each injection from different groups of mice. Cells from three animals at each time point were pooled and populations of immune cells were measured by flow cytometry. We found in preliminary experiments that significant changes in cell populations did not occur in the 24 h after the first injection. However, as illustrated in Fig 8, populations of most cells types increased over the period of treatment. In particular, populations of DCs (CD11c^+^), NK cells (NK1.1^+^), T cells (CD3^+^), T_H_ cells (CD4^+^), cytotoxic T cells (CD8^+^), and B cells (CD19^+^) increased several-fold, including those that expressed the activation marker CD69. Memory B cells that expressed CD73, CD80 and CD273 increased significantly.

## Discussion

Our data support peptide svH1C as a mimetic of Neu5Ac-Gal/GalNAc or Neu5Ac-Neu5Ac. The CD spectra of svH1C suggest that the arms of the peptide aggregate at a sufficiently high concentration. The aggregation may reflect the PxxP sequence in the peptide, which is similar to collagen. Indeed, the CD spectrum of gelatin obtained under the same conditions had a minimum at 238 nm. Interestingly, the negative signal at 234 nm in the spectrum at 100 μM is fortuitously similar to that of sialic acid lactones, including the sequence Neu5Ac-Gal [65], but this minimum disappeared as the peptide was diluted. The tetravalent structure of the peptide provides a high local concentration within the sphere of a single molecule, but this feature is not sufficient to produce a CD signal. Analysis of solutions of the peptide by NMR spectroscopy did not reveal a definite structure, which suggested that the ‘arms’ are extended and have a high degree of flexibility. At the low concentrations used for the binding studies, the peptide apparently achieved a random structure. Evidence for a high degree of flexibility does not provide information on a specific binding mode. It simply provides an idea for the plasticity of the peptide and its tendency to explore conformational space and adopt the preferred conformation for partner recognition and interaction. The flexibility of the peptide allows it to interact with multiple members of the siglec family of receptors.

The flexibility of the arms of the tetravalent structure was retained in binding assays. When added to streptavidin-coated wells, the biotinylated peptide was anchored at the C-terminus, which allowed full flexibility of the arms [29]. Likewise, when the receptors were first bound to wells via a Fc domain or a His extension, the interaction with peptide occurred in solution and the peptide that remained bound after extensive washes was then detected by streptavidin binding to the C-terminal biotin tag.

Because svH1C bound to plant lectins specific for Neu5Ac-Gal sequences, we tested the ability of the peptide to bind to recombinant human lectin-type receptors that bind ligands containing these glycans. The siglec family is the most prominent among the receptors for which sialylated glycans serve as ligands. Differences in binding of svH1C were found among those tested, with strong binding to Siglec-1, -5, -7, -11, -9 and -14 but less with Siglecs-2, and -3 in PBS. The low binding detected with several siglecs was sensitive to the conditions of the assay. It is likely that svH1C binds to Siglecs-2 and -3 but that the interaction does not survive the extensive washes with buffer. However, binding to other lectin-type receptors such as CLEC9a, CLEC10a and DC-SIGN was not promoted by use of other buffers or inclusion of Ca^2+^. These observations suggest that the standard assay was capable of discriminating within a range of avidity and specificity. A common feature between Siglec-5 and -14 is their preference for a terminal Neu5Ac(α2,8)Neu5Ac or Neu5Ac(α2,6)GalNAc sequence [17]. Siglec-1 is specific for terminal Neu5Ac(α2,3)Gal, which is also a ligand for NKG2D [27]. The inhibition of binding by the multivalent fetuin suggests that the peptide interacts with the glycan binding sites of these receptors.

The abundance of sialylated glycans on the cell surface, estimated to provide a local Neu5Ac concentration of 110 mM on B cells [13,63], and the many receptors that bind Neu5Ac-containing glycans call into question whether a drug can bind with sufficient avidity and specificity to achieve a biological response. Cell-surface proteins are heavily glycosylated, which allows siglecs to bind sialylated proteins on the same cell (*cis*) or on neighboring cells (*trans*) and serve as adhesion proteins [63,64]. Siglecs tend to be specific for the linkage between terminal Neu5Ac and the penultimate sugar, usually Gal, which suggests a certain rigidity of the binding site and the ligand. A flexible peptide can therefore adjust its confirmation to fit into a rigid binding site, consistent with the Monod-Wyman-Changeux hypothesis [46]. The siglecs bind their natural glycan ligands with relatively low affinity, with K_D_ values from the 100 to 400 μM range [9,66] into the mM range [23]. Siglec-2 (CD22) binds to CD45, a heavily glycosylated and abundant cell surface protein, with a K_D_ of 117 μM [66]. The kinetics of binding, with a rapid off rate, suggest the probability of easy exchange of ligands on siglecs [63,64,66].

Most siglecs are inhibitory receptors containing an ITIM [9–12] and express full activity when a ligand is bound [16]. As documented with Siglec-2 on B cells, a functional ligand binding site is required for tyrosine phosphorylation, recruitment of the phosphatase SHP-1, and inhibition of B cell activation [67]. However, Kelm et al. [68] showed that binding of a sialoside, methyl-α-9-*N*-(biphenyl)-4-carbonyl)-amino-9-deoxy-Neu5Ac (BPC-Neu5Ac), which bound Siglec-2 with an IC_50_ = 4 μM after cells were treated with neuraminidase, reduced tyrosine phosphorylation and attenuated the receptor’s inhibitory activity. This work was followed with synthesis of additional sialoside derivatives, which bound human Siglec-2 with an IC_50_ as low as 2 nM and also attenuated its inhibition of activation of B cells by anti-IgM [69].

As shown in Fig 7, a rapid decrease of phosphorylated tyrosine in siglecs was detected within a few minutes of treatment with svH1C, with the greatest reduction on Siglec-3, -5 and -7. Phosphorylated Siglec-2 and -9 were abundant in the untreated PBMC cultures, but significant dephosphorylation also occurred after addition of svH1C. These siglecs are expressed by several different immune cell types [11,12]. FcRH4/IRTA1 and NKp46, which had less reduction in phosphorylation, are expressed on memory B cells and NKT cells, respectively [12,63,70]. The decrease in the active forms of siglecs, and the receptor LAIR-1, which is expressed on most mononuclear leukocytes and contains two ITIMs [71], suggest a reduction of inhibitory activities within monocytes. CD229, member 3 of the SLAM (signaling lymphocyte activation molecule) family of immune receptors, contains two cytoplasmic ITSM motifs and can initiate positive or negative signals [72]. CD229 is expressed most strongly by CD4^+^ and CD8^+^ T cells, to a lesser extent by B cells and slightly by NK cells. Ligation of CD229 or the T cell receptor (TCR) by specific antibodies leads to phosphorylation of CD229 and recruitment of Grb2, which favors internalization [73,74]. The dramatic loss of phosphorylated CD229 and other receptors may result from activation of phosphatases. The effects are clearly induced by interactions of svH1C on the cell surface, but whether changes in phosphorylated receptors other than siglecs are direct or indirect effects of svH1C is not known.

NKG2D lacks a signaling motif in its cytoplasmic domain and functions as an activating receptor in association with adaptor proteins, DAP10 and DAP12 [75]. In addition to NKG2D, NK cells express other activating cytotoxicity receptors such as NKp30, NKp44 and NKp46. These receptors bind to tandemly multivalent heparan sulfate/heparin sequences with K_D_ values of 0.3 to 2 μM [76]. Similarly to NKG2D, NKp46 also binds multivalent sialyl-Lewis^X^ with a K_D_ of approximately 0.6 μM [77]. The native *in vivo* ligands for NKG2D are divergent proteins, MIC-A/B, ULBP, Rae-1 or H60, which bind the receptor with K_D_ values of approximately 1 μM [24–26]. In comparison with these data, svH1C binds to NKG2D with a similar K_D_ value, 0.6 μM, as determined by microcalorimetry. The avidity of svH1C to NKG2D was also estimated by the inhibition of binding by fetuin, a glycoprotein with multiple glycans with terminal Neu5Ac-Gal sequences, which yielded a binding constant of near 1 μM (Fig 4).

Multivalent structures achieve several orders of magnitude greater avidity than single glycans [21–23,41]. Thus, these results lead to the conclusion that svH1C is an effective glycan mimetic and binds with avidities equal to or greater than natural multivalent glycan structures. svH1C or similar glycan mimetics are readily synthesized, non-toxic *in vivo*, and may find practical application as immunomodulatory therapeutic agents. Because the data suggest that the activity of svH1C leads to attenuation of inhibitory receptors and apparent stimulation of immune cells *in vivo*, this peptide expresses characteristics of checkpoint inhibitors that are important in immunotherapy [78,79].

## Supporting Information

S1 Table ^1^H Chemical shifts (ppm) of the [NPSHPLSGGGGS]K svH1C segment at 298K (H_2_O/D_2_O 90%/10% v/v, phosphate buffer, pH 7.0). (DOCX)

S1 Fig ^1^H 400 MHz NMR of 1 mM svH1C on a 16.0204 ppm spectrum recorded in H_2_O/D_2_O (90%/10% v/v, T = 298K, phosphate buffer, pH 7.0). (TIF)

S2 Fig Binding of svH1C to NKG2D, assayed by microcalorimetry. (TIF)
