# Supplementary material for: A Peptide Mimetic of 5-Acetylneuraminic Acid-Galactose Binds with High Avidity to Siglecs and NKG2D
Source: PLoS One. 2015 Jun 25;10(6):e0130532. doi: 10.1371/journal.pone.0130532 (PMC4482410; doi:10.1371/journal.pone.0130532)
Supplement: S1 Table — (DOCX) [file pone.0130532.s001.docx]

| Residue | | HN | Hα | Hβ | other |
| --- | --- | --- | --- | --- | --- |
|  |  |  |  |  |  |
| 1 | Lys | 8.113 | 4.147 | 1.703/1.637 | Ηγ 1.271, Ηδ 1.432, Ηε 3.108,  Hζ 7.859 |
|  | Lys | 8.227 | 4.246 | 1.745/1.654 | Ηγ 1.277, Ηδ 1.436, Ηε 3.132  Ηζ 7.943 |
|  | Lys | 8.181 | 4.150 | 1.712/1.646 | Ηγ 1.275, Ηδ 1.436, Ηε |
| 2 | Ser | 8.129 | 4.323 | 3.783 | ----------------------- |
|  | Ser | 8.206 | 4.398 | 3.825 | ----------------------- |
|  | Ser | 8.323 | 4.315 | 3.750 | ----------------------- |
| 3 | Gly | 8.167 | 3.798 | -------- | ----------------------- |
|  | Gly | 8.268 | 3.939 | -------- | ----------------------- |
|  | Gly | 8.330 | 3.927 | -------- | ----------------------- |
|  | Gly | 8.377 | 3.954 | -------- | ----------------------- |
| 4 | Leu | 8.554 | 4.344 | 1.623 | Ηγ 1.568, δCH_3_ 0.885/0.827 |
| 5 | Pro | -------- | 4.444 | 2.284/1.960 | Ηγ 1.861, Ηδ 3.696/3.618 |
|  | Pro | -------- | 4.374 | 2.228/1.965 | Ηγ 1.860, Ηδ 3.676/3.640 |
| 6 | His | 7.994 | 4.511 | 2.955 | Ηδ , Hε , Hζ |
| 7 | Asn | 8.139 | 4.861 | 3.085/2.997 | δΝΗ_2_ |
|  |  |  |  |  |  |

Eggink et al., Table S1
