# Supplementary figures and images for: A Peptide Mimetic of 5-Acetylneuraminic Acid-Galactose Binds with High Avidity to Siglecs and NKG2D
Source: PLoS One. 2015 Jun 25;10(6):e0130532. doi: 10.1371/journal.pone.0130532 (PMC4482410; doi:10.1371/journal.pone.0130532)

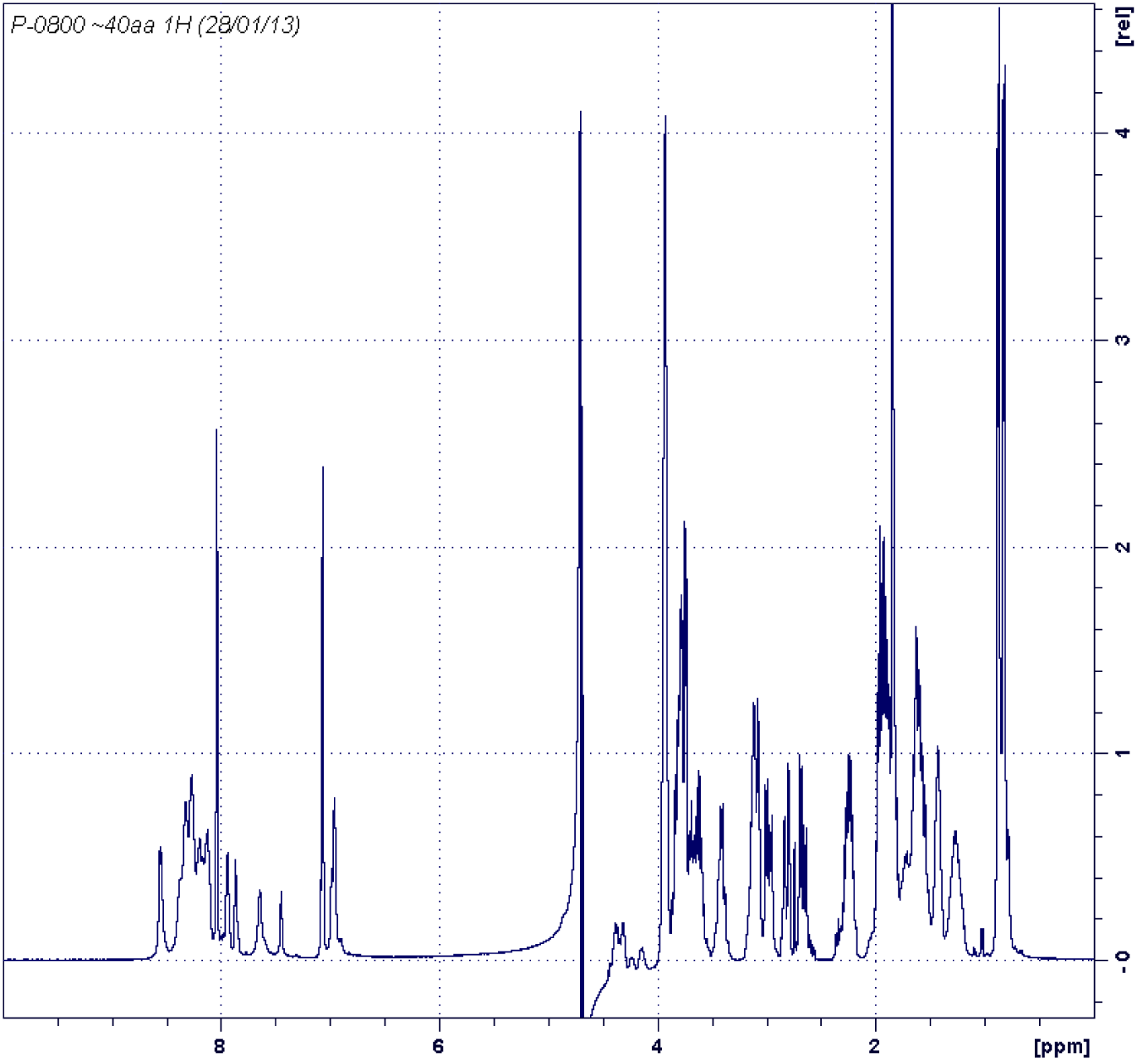

Supplement: S1 Fig — (TIF) [file pone.0130532.s002.tif]

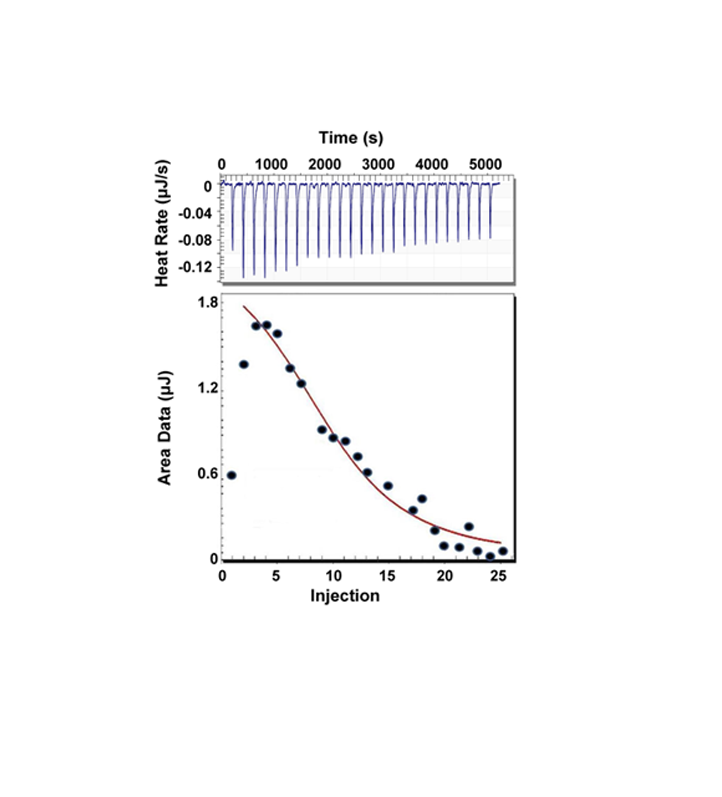

Supplement: S2 Fig — (TIF) [file pone.0130532.s003.tif]
